# Colistin Resistance in Bacteria: Current Updates on Mechanism of Action and Combating Strategies

**DOI:** 10.1155/cjid/8603173

**Published:** 2026-04-21

**Authors:** Indu Singh, Anil Kumar, Indira Kumari Verma, Divakar Sharma, Amritesh Kumar Singh

**Affiliations:** ^1^ Department of Pharmacology, Graphic Era Hill University, Dehradun, Uttarakhand, India, gehu.ac.in; ^2^ Department of Biotechnology, Graphic Era (Deemed to be) University, Dehradun, Uttarakhand, India; ^3^ Department of Microbiology, Veer Chandra Singh Garhwali Government Institute of Medical Science and Research, Srinagar, Uttarakhand, India; ^4^ Department of Microbiology, Shri Kalyan Government Medical College, Sikar, Rajasthan, India; ^5^ Autonomous State Medical College, Kushinagar, Uttar Pradesh, India

**Keywords:** colistin, Gram-negative bacteria (GNB), horizontal gene transfer, mobile colistin-resistant genes (*mcr*), polymyxin

## Abstract

The rapid development of colistin‐resistant bacteria is the biggest global health issue since colistin is referred to as a ‘last‐hope resource’ against life‐threatening infections caused by GNB (Gram‐negative bacteria). Injudicious and excessive colistin application in clinical, agricultural and veterinary practices, as well as plasmid‐mediated horizontal gene transfer and chromosomal mutation, commonly contribute to colistin resistance. The coexistence of the *mcr*‐1 gene with *mcr* variants, other intrinsic genes and other mechanisms might provide additional resistance strength against colistin. To prevent the emergence of colistin‐resistant strains and preserve the therapeutic efficacy of existing antibiotics, we must expand our deep understanding of colistin‐resistant strains at the molecular level. This critical review discusses the mechanism of action, various bacterial resistance mechanisms, the presence of *mcr* genes in bacterial plasmids, hetero‐resistance, biofilm formation, the horizontal transfer or dispersion of colistin‐resistant genes and combating strategies. Our synthesis underscores the need for strategically aligning mechanistic knowledge with practical therapeutic solutions to combat escalating colistin resistance and inform future medical decision‐making and research planning.

## 1. Introduction

The emergence of antimicrobial resistance poses a major public health risk worldwide, causing millions of deaths every year owing to the injudicious and excessive use of antibiotics [1]. Bacteria undergo multiple genetic changes, either chromosomal or plasmid, that develop resistance to a broad class of antibiotics [2]. Clinicians confront challenges in treating bacterial‐induced infectious diseases owing to multidrug resistance (MDR) and a lack of appropriate antibiotics. Drying up the novel antibiotic findings against these superbugs forces the clinician to utilize the older class of antibiotics called polymyxin, specifically colistin, which has been considered a ‘last‐resort’ antibiotic [3]. Polymyxin is a class of non‐ribosomal cyclic antibiotics with different kinds of compounds referred to as A, B, C, D and E. The usage of polymyxin‐B and colistin has re‐emerged in clinical practices as they are effective against resistant Gram‐negative bacteria (GNB) [4, 5]. Colistin (poly‐cationic oligopeptide) belonged to polymyxin‐E and was identified in 1947 in Japan from a culture of Gram‐positive bacteria called *Paenibacillus polymyxa sub-species colistinus*, and it is effective against dreadful infections caused by aerobic GNB, *Escherichia coli, Acinetobacter baumannii, Klebsiella pneumoniae* and *Pseudomonas aeruginosa* [6]. Chromosomal alteration in GNB interferes with the electrostatic interaction between lipid‐A molecules found in the outer membrane (OM) of bacteria and colistin, resulting in colistin resistance [7]. Some bacteria possess intrinsic colistin resistance mechanisms (*Serratia merecscens*, *Proteus mirabilis*, *Burkholderia* spp., *Morganella morganii*, *Providencia* spp., *Vibrio cholerae*, *Chromobacterium* spp., *Neisseria* spp., *Brucella* spp. and *Legionella* spp.) and some acquired resistance mechanisms (*E. coli*, *P*. *aeruginosa*, *A*. *baumannii*, *Salmonella* spp., *Klebsiella* spp. and *Enterobacter* spp.) [8]. Multiple mechanisms (modification of lipopolysaccharide (LPS), capsule formation, reduction of overall negative charge on LPS and excessive production of efflux pumps) are employed by bacteria to mitigate the effect of colistin [9, 10]. Concern has been raised about the usage of colistin due to the escalating evolution of resistant GNB because of injudicious and over‐prescription. The development of novel traits in GNB reflecting colistin resistance mitigates the use of colistin in clinical practices. This review explores colistin’s mode of action, the bacterial resistance mechanism, the *mcr* genes on bacterial plasmids and the dispersion of colistin‐resistant genes that lead to the emergence of resistant GNB strains. This review synthesizes a perspective that is distinct from previous literature by bringing together the molecular foundations of colistin resistance along with a structured examination of current and emerging mitigation strategies. Rather than treating mechanisms and interventions as separate topics, we integrate chromosomal alterations, plasmid‐mediated pathways, membrane remodelling, biofilm‐associated tolerance and hetero‐resistance into a single conceptual narrative that directly links how resistance arises and to be countered. We further highlight therapeutic developments, ranging from re‐engineered drug combinations, repurposed agents, nano‐scale platforms, peptide‐based antimicrobials, photodynamic approaches, bacteriophage tools and CRISPR‐driven technologies, which discuss their relative promise and translational progress. However, this article focuses specifically on mechanistic and therapeutic dimensions; other areas, such as global epidemiology and diagnostic advances, are summarized but intentionally not explored in depth so that the review maintains a clear and coherent scope.

### 1.1. Colistin and Its Mechanisms of Action

Colistin is an amphipathic polycationic cyclic peptide that consists of two components (one is a hydrophobic core and the other a fatty acid chain) [11]. The fatty acid chain is attached to the core region by a tripeptide bridge. Colistin exhibits a positive charge attributed to the presence of diaminobutyric (Dab) acid in its core region, which is critical for its antibacterial activity (Figure 1) [12]. There are three key characteristics of cationic Dab residue that provide its antibacterial effect: (i) cationic side chain group, (ii) two–CH2 (methylene) group on Dab side chain and (iii) specific order of Dab residues [13, 14]. Colistin is used in clinical practices in two forms: (i) active form (CS‐colistin sulphate) and (ii) pro‐drug form (CMS‐colistimethate sodium or colistin methanesulphonate) [11]. CMS exhibits lower toxicity compared to CS and is administered via parenteral routes like intramuscular (IM), intravenous (IV) and nebulization routes, whereas CS is administered through oral and dermal routes [15]. The pro‐drug structure is different from the typical colistin molecule because methanesulphonate is attached to the Dab [16].

FIGURE 1(a) Structure of colistin. (b) Structure of colistin methanesulfate (CMS).

**Figure:**
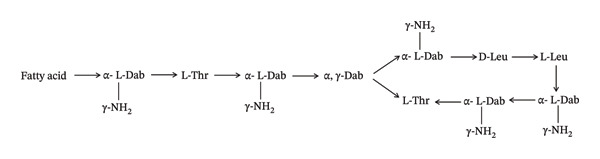
(a)

**Figure:**
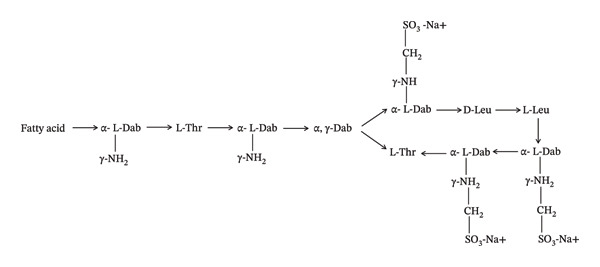
(b)

The lipid‐A component of LPS of the OM of GNB provides stability and cations (Ca^++^ and Mg^++^ ions) that cross‐link adjoining lipid‐A molecules and provide rigidity to the OM [17, 18]. Colistin disrupts the OM by dislodging the Ca^++^ and Mg^++^ ions via electrostatic exchange between the Dab and phosphate group of lipid‐A components of LPS [19]. Colistin exhibits two types of modes of action to induce bacterial cell death: (i) primary (membrane lysis) and (ii) secondary (inhibition of respiratory enzyme, vesicle–vesicle interaction mechanisms and free radical generation mechanisms). Various mechanisms have been explored through which colistin induces bacterial cell death.

i. Conventional or direct antimicrobial mechanisms: Membrane lysis is most commonly attributed to colistin. The cationic Dab interacts with the phosphate head of the lipid‐A component and dislodges the Ca^++^ and Mg^++^ ions from the phosphate head, disrupting LPS (Figure 2(a)) [20]. Further, colistin expands the destabilization of LPS by introducing a fatty acyl chain or D‐Leu6‐l‐Leu7 segment, inducing swift self‐uptake of colistin through the destabilized portion of OM [21, 22]. Ultimately, the inner membrane (IM) of the phospholipid bilayer thins, followed by loss of integrity, leading to cell lysis (Figure 2(b)) [10, 20].
ii. Inhibition of respiratory enzyme: Recent research has discovered a mechanism that targets the respiratory enzyme, specifically type‐II NADH oxidoreductase (Figure 2(c)) [23]. There is no involvement of type‐II NADH oxidoreductase in transporting H^+^ ions across membranes [24]. Colistin targets this enzyme, triggering its consumption, which leads to alteration in the bacterial electron transport chain (ETC), eventually culminating in bacterial cell death [25]. Colistin inhibits the NADH oxidase enzyme in *Bacillus* species while inhibiting the malate‐quinone oxidoreductase enzyme and the NADH dehydrogenase enzyme in *Mycobacterium smegmatis* [6].
iii. Generation of hydroxyl (OH^∗^) radical: Evidence from past studies showed that colistin induced death via the generation of ROS (reactive oxygen species) that causes oxidative stress (Figure 2(d)) [19, 26]. In particular, the OH^∗^ radical damages proteins, lipids and DNA [27]. Colistin induces the production of O^2-^, which is converted into H_2_O_2_ by the superoxide dismutase (SOD) enzyme, resulting in the generation of Fe^3+^ ions, which trigger the Fenton reaction and culminate in the production of OH^∗^ [19]. Previous evidence indicated that *A. baumannii* and *Escherichia coli* strains are killed through OH generation via the Fenton reaction but do not exhibit a killing effect in polymyxin‐resistant strains [27].
iv. Anti‐endotoxin mechanisms: LPS of GNB is referred to as endotoxin [15]. Upon infection, endotoxin triggers the production of inflammatory cytokines such as TNF‐α and interleukin‐8 (IL‐8), eventually leading to immune shock (Figure 2(b)) [28, 29]. Colistin neutralizes the endotoxin activity by disrupting the LPS via electrostatic interaction with the phosphate head of the lipid‐A component of LPS [28].
v. Vesicle–vesicle contact mechanisms: An alternative mechanism employed by colistin is called vesicle–vesicle contact mechanisms [30, 31]. Colistin adjoins the outer leaflet of IM to the inner leaflet of OM, leading to phospholipid exchange between vesicles, resulting in loss of a specific phospholipid composition (Figure 2(e)) [31]. As a result, osmotic equilibrium is upset, which ultimately causes cell lysis [19].

FIGURE 2Mechanisms of colistin‐mediated antibacterial activity. (a) Direct anti‐bacterial activity of colistin. (b) Colistin‐mediated respiratory enzyme inhibition. (c) Colistin‐mediated OH^∗^ radical death mechanism. (d) Anti‐endotoxin activity of colistin. (e) Vesicle–vesicle death mechanisms induced by colistin.

**Figure:**
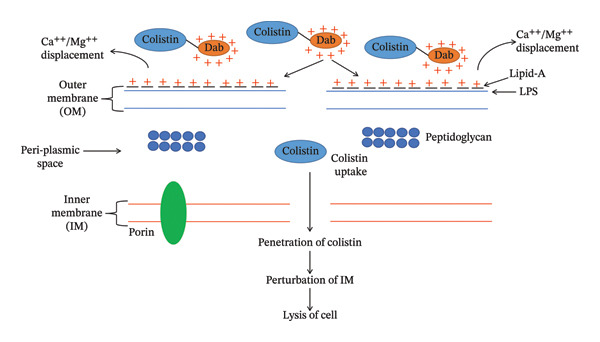
(a)

**Figure:**
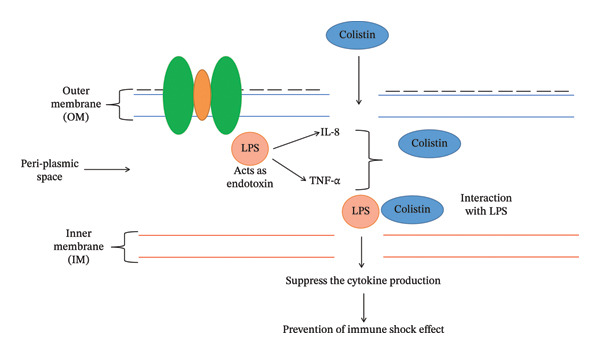
(b)

**Figure:**
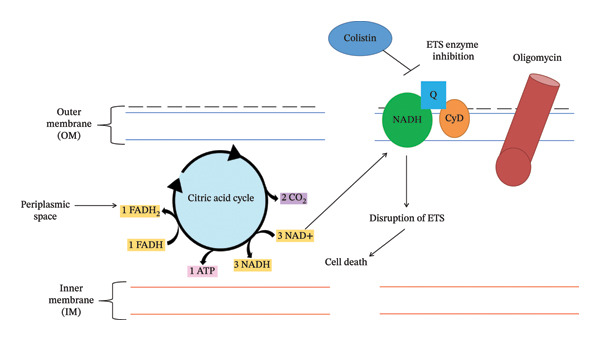
(c)

**Figure:**
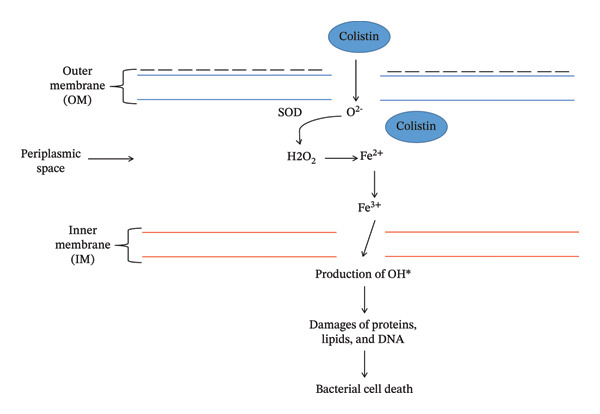
(d)

**Figure:**
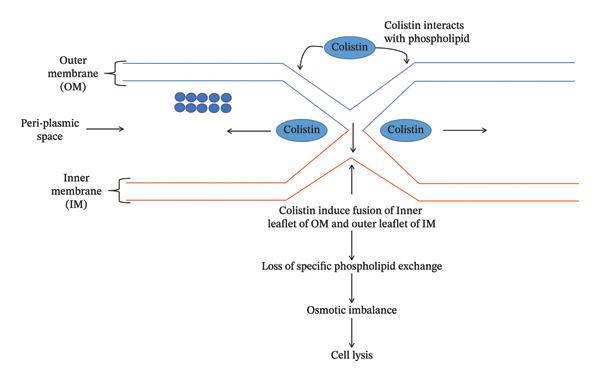
(e)

## 2. Clinical Toxicity of Colistin

Clinicians are discouraged from using colistin because of its toxic effects. Nephrotoxicity and neurotoxicity are linked with colistin application in clinical practices [32]. Compromised kidney function and marginally enhanced serum creatinine (0.3 ± 0.8 mg/dL) levels have been reported with colistin treatment [33]. During a study conducted in Taiwan, nephrotoxicity was observed in colistin‐treated patients [34]. In some patients, nephrotoxicity recovered while in others, short‐term haemodialysis (2–3 weeks) was required. Colistin enhanced the toxic effects of other drugs like aminoglycosides when used together [35]. In people with renal impairment, colistin raised the serum creatinine level more than in healthy individuals [36]. A study conducted on American patients revealed that 21% of patients had their therapy stopped because of nephrotoxicity, while 45% of patients experienced nephrotoxicity as a result of CMS medication [37]. Additionally, they noted that patients who received treatment for longer than 14 days were more susceptible to nephrotoxicity. One study conducted in South Korea observed that 20% of patients required kidney replacement therapy following colistin medication, while 31.7% of patients developed nephrotoxicity [38]. One clinical study reported marginal changes (0.2 mg/dL) in serum creatinine levels after colistin medication, while 14.3% of patients developed nephrotoxicity [39].

## 3. Mechanism of Colistin Resistance

Excessive colistin usage triggers resistance mechanisms in GNB under selective evolutionary pressure that creates a global health threat because of antibiotic scarcity to cope with GNB infections [40]. Some GNBs possess intrinsic resistance mechanisms, while some acquire resistance mechanisms [8]. GNB employs multiple mechanisms: (i) modification in LPS, (ii) capsule formation, (iii) high expression of efflux pump, (iv) hetero‐resistance and (v) biofilm‐induced resistance.

i. LPS modification in colistin‐resistant bacteria: LPS structure modification occurred in three ways: (a) chromosomal‐induced LPS modification, (b) plasmid‐induced LPS modification and (c) complete loss of LPS structure.
  a. Chromosomal‐mediated LPS modification: A reduction in the overall negative charge perturbs the interaction between LPS and colistin, resulting in resistance to colistin [41]. Multiple operons and intrinsic genes of bacterial chromosomes are involved in LPS alterations: (1) two‐component regulatory system (*PhoPQ*, *PmrAB* and *crab*), (2) negative regulatory genes (*mgr-B*) and (3) product of operons (*arnBCADTEF* operon) and genes (*pmrC* and *pmrE*) provide the addition of positively charged compounds, either pEtN (phosphoethanolamine) or L‐Ar4N (4‐amino‐4‐deoxy‐L‐arabinose).
   pEtN and L‐Ar4N are added to lipid‐A by PmrC, a protein with intrinsic pEtN transferase activity, and PmrE proteins encoded by the *pmrABC* and *arnBCADTEF* or *pmrHIJKLM* operons, respectively [42–44]. PmrB functions as a biological sensor that phosphorylates PmrA in response to acidic pH and Fe^++^ in the surrounding environment [45, 46]. Consequently, the *pmrE* gene, *pmrHFIJKLM* and *pmrABC* operons are activated by PmrA, resulting in LPS modification [47]. LPS modification by the addition of L‐Ar4N is considered a prevalent resistance mechanism among various GNBs. *A*. *baumannii* employs a non‐conventional colistin resistance mechanism (addition of galactosamine to lipid‐A) because it lacks genes for L‐Ar4N synthesis [48]. A mutation of *PmrA/PmrB* leads to an increase in the expression of *pmrE*, *pmrHFIJKLM* and *pmrABC* operons, which lead to colistin resistance after LPS modification [49, 50]. In addition to *PmrA/PmrB*, the *pmrHFIJKLM* operon is also activated by the PhoP/PhoQ regulatory system [2, 51]. PhoP/PhoQ‐mediated *pmrHFIJKLM* operon transcription occurs under sub‐lethal polymyxin concentrations or low Mg^++^ growth conditions [52, 53]. Upon sensing these conditions, PhoQ activates PhoP by phosphorylating it, which stimulates the *pmrHFIJKLM* operon transcription [12]. On activation, PhoP also indirectly activates PmrA through phosphorylation of the PmrD protein, followed by synthesis and addition of PEtn to lipid‐A [2, 51]. Genetic alteration evidence identified PhoP/PhoQ mutation in *E. coli* and *K. pneumoniae,* which results in acquired colistin resistance [8, 54, 55]. Evidence on *P*. *aeruginosa* found that either individual or combined deletion of the ColRS/cprRS results in colistin resistance in *PhoQ* mutants, indicating that LPS modification is regulated by *PhoP/PhoQ*, as well as the ColRS/cprRS regulatory system, providing an additional level of colistin resistance [56]. Accumulating reports observed that mutations in IS (insertion sequence‐*IS5-like, IS10R, ISKpn14, IS1F and ISKpn13*) element *mgrB* genes culminate in the activation of PhoQ, followed by LPS modification, suggesting that *mgrB* negatively regulates the kinase activity of PhoQ protein [57–59]. Clinical research showed that colistin resistance caused by *mgrB* mutations is a common mechanism in *K*. *pneumoniae* [59]. Genetic studies indicated that the *CrrAB* mutation contributed to another level of colistin resistance in strains of *K*. *pneumoniae* [60, 61]. Reports showed that *CrrAB* inactivation was attributed to the activation of *pmrE* and *pmrC* genes and the *pmrHFIJKLM* operon by increasing the expression of the *pmrAB* operon [59, 61]. Several other genes (*eptA*, *eptB* and *eptC*) are also involved in LPS modification, contributing to colistin resistance [62]. Report on *E. coli* showed that an increased amount of regulatory protein expression is associated with colistin resistance [63]. One study on *A*. *baumannii* observed a high expression of EptA protein and colistin resistance in *eptA* mutants [64].
  b. Plasmid‐mediated LPS modification: Research around the world was revolutionized by the first study that showed a correlation between the plasmid‐residing *mcr* gene and PEtN transferase expression [65]. Other *mcr* (*mcr2-10*) gene families with > 100 variants among GNB were reported by several investigations [66–69]. Because of its ease of spread both within and across species, colistin resistance brought on by the *mcr* gene is a serious concern. Colistin resistance is caused by the *mcr-1* gene, which encodes PEtN, modifying LPS to weaken its interaction with LPS [70]. The *mcr*‐1 gene provides additional antibacterial activity by inactivating lysozyme action [71]. Several studies demonstrated the *mcr*‐*1* gene–mediated colistin resistance in various *E. coli* isolates, particularly from chicken and pigs [69]. The MCR‐1 protein’s catalytic domains have been revealed [72], but the substrate‐binding site has not yet been investigated. Our understanding of the molecular mechanisms underlying horizontal gene transfer is limited by scarce knowledge regarding *mcr* gene–mediated colistin resistance.
  c. Loss of LPS structure: Loss of LPS core or lipid‐A molecule contributes to colistin resistance observed in *A. baumannii* [11]. It was demonstrated in an artificially induced mutational study that mutations in genes (*lpsB*, *lpxD*, *lpxC* and *lpxA*) responsible for lipid‐A synthesis caused colistin resistance in *A. baumannii* [73]. Studies reported the insertion and deletion of biosynthesis genes in isolates and mutants, leading to the formation of truncated proteins contributing to colistin resistance [74–76]. According to clinical studies on *A. baumannii*, the insertion of IS elements causes the *lpxD* and *lpxC* genes to lose their function, contributing to colistin resistance [77, 78]. In clinical isolates, LPS‐deficient strains have not been identified due to null survival [79].
ii. Capsule formation: Capsule formation is another resistance mechanism observed in *K. pneumoniae* [42, 80]. Investigations reported that *Rcs* and *Cpx* genes were involved in capsule formation [81]. One report on *K. pneumoniae* showed a correlation between enhanced CPS (capsular polysaccharide) expression and resistance to polymyxin‐B [82]. They also mentioned that CPS protected those strains that lack O‐antigen from polymyxin B. Additionally, a correlation between the *ugd* gene and capsule synthesis was noted [83, 84]. The number of CPS layers surrounding GNB determines the resistance [5].
iii. Upregulation of efflux pump: Some studies demonstrated an association between GNB’s colistin resistance and increased efflux pump expression [85, 86]. There are two genes (*AcrAB* and *KpnEF*) in *Enterobacteriaceae* that are known to be involved in the synthesis of efflux pumps [87]. Enhanced sensitivity to multiple colistin peptides has been reported in *KpnEF* mutants [87]. Studies on different GNB strains (*Salmonell*a, *E. coli* and *K. pneumoniae*) demonstrated that *AcrAB* up‐regulation contributes to colistin resistance [88–90]. The function of the *EmrAB* system in colistin resistance is supported by data from in vitro research on *emrAB* mutants [91]. Increased expression of genes (*macAB*, *adeC*, *mexB*, *emrB* and *adeI*) that encode components of the efflux pump has been reported to contribute to colistin resistance in *A. baumannii* [92]. The application of an antibiotic that targets ribosomes resulted in colistin resistance in *P. aeruginosa* due to the up‐regulation of MexXY efflux pumps [93]. As a result of heterogeneity in *MexXY* expression, *P. aeruginosa* displays variable resistance patterns [94]. Studies based on efflux pump inhibitors on *Salmonella maltophilia*, *P. aeruginosa*, *A. baumannii* and *K. pneumoniae* proved the role of efflux pumps in colistin resistance [95].
iv. Hetero‐resistance: It is a phenotypic resistance mechanism in which the subpopulation is more resistant to antibiotics than the main population [96]. Colistin resistance can be developed through hetero‐resistance [6]. Hetero‐resistance bacteria produce sub‐populations with differential degrees of colistin‐resistance [97]. Hence, in the presence of colistin, it induces the amplification of colistin resistance sub‐population [6]. The hetero‐resistance mechanism is found to be more prevalent in MDR *K. pneumoniae* and *A. baumannii* [98]. Investigations revealed that colistin hetero‐resistance occurred due to mutations in *lpxD*, *lpxA* and *lpxC*, which are responsible for lipid‐A biosynthesis [73, 99]. Populations of mutant hetero‐resistance are found to be stable while the main population re‐emerges in infected patients [99].
v. Biofilm‐mediated colistin resistance: Biofilm is an adaptation or protective strategy to the surrounding environment, referring to organized bacterial populations formed by extracellular DNA, protein and polysaccharides [100, 101]. Past investigations on GNB indicated that GNB infection and biofilm formation are correlated [102, 103]. The presence of biofilms makes conventional antibacterial drugs difficult to penetrate [104–106]. A biofilm appears to be a favourable environment for the dissemination of antibiotic‐resistance genes (ARGs) across strains [107, 108]. Investigation reported the correlation between enhanced *PhoQ* expression and biofilm formation contributing to colistin resistance [109]. Biofilm‐mediated colistin resistance has been observed in *A. baumannii* and avian pathogenic *E. coli* [109]. A study on a colistin‐resistant *A. baumannii* strain reported that CsuC and CsuA/B proteins are involved in biofilm formation [110].

## 4. Distribution of Colistin Resistance Genes

Health risks arise from the lack of therapeutic drugs against GNB and the rapid dissemination of plasmid‐linked *mcr* genes. Rapid dissemination of the *mcr* gene occurs throughout the world owing to (i) molecular flexibility of the *mcr* gene, specifically the *mcr-*1 gene, (ii) conjugative property of plasmid backbone, (iii) presence of IS element upstream of *mcr* genes, (iv) horizontal gene transmission (transformation, conjugation and transduction and (v) excessive application of colistin in veterinary practices [111–114].

Several kinds of plasmids (IncHI2, IncY, IncP, IncI2, IncHI1, and IncX4) are involved in the dissemination of *mcr-1* genes, of which IncX4 and IncI2 are the most common plasmids [113]. Several studies discovered that the *mcr* genes were also found on the IncFI, IncFII, IncFIB, IncK2, IncF, IncN and IncQ plasmids [115–117]. An investigation found that one and two copies of the ISApl1 IS‐element flanked the *mcr-1* gene in IncHI2/IncI2 and IncX4 plasmids, respectively, which is responsible for transposon activity [65, 118]. Loss of the IS element, either through non‐homologous recombination or the transposition process, leads to stabilization and mobility of the *mcr-1* gene that causes colistin resistance [114, 119]. A similar mechanism is responsible for the dissemination of other *mcr* genes, such as IS1595 IS‐element flanking the *mcr* gene upstream [117]. TnAS2, ISKpn6 and Tn3‐family transposons flank the *mcr-3, mcr-4* and *mcr-5* genes, respectively [68]. According to recent studies, plasmids carrying *mcr* genes also carry other ARGs, including *KPC-3*, *NDM-5*, *NDM-1* and *KPC-2*. Co‐existence of colistin resistance genes and other ARG genes on plasmid disseminates simultaneously through the conjugation process, resulting in inter‐ and intra‐species dissemination of ARGs [120–122]. The dissemination of other resistance determinants along with colistin resistance genes creates a hurdle to treating/managing GNB‐induced infection in clinical practices.

### 4.1. Plasmid‐Mediated *mcr* Genes

**FIGURE 3:**
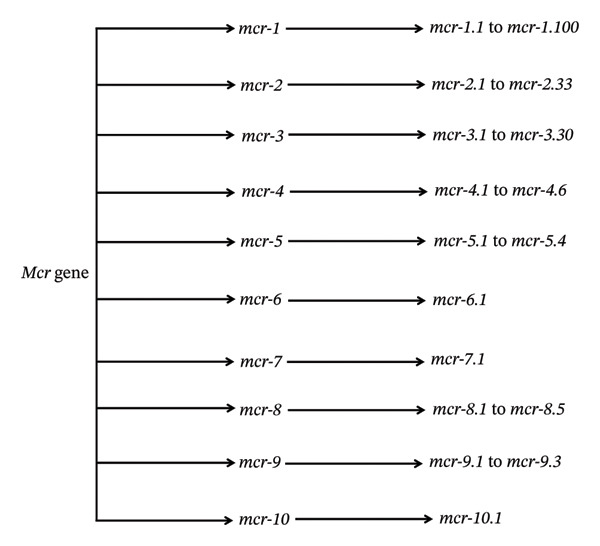
Plasmid‐linked mcr genes and their variants that contribute to horizontal gene transmission.

*mcr* genes are found on plasmids responsible for the rapid dissemination of colistin resistance GNB across the globe. It has been found that 10 types of *mcr* genes (*mcr-1-10*) exist on different kinds of plasmids with 100 variants of these genes (Figure 3) [123, 124].

i. *mcr-1* gene: The first report of *mcr-1* appeared in 2015, yet retrospective investigations later traced the gene to bacterial samples from the early 1980s, revealing a prolonged period of undetected dissemination preceding its official discovery [65, 125, 126]. The *mcr-1* gene has 25 variants, and the size of the *mcr-1* gene is 1626 bp [1]. Among all *mcr* genes, the *mcr-1* gene is highly prevalent worldwide. *Enterobacteriaceae* are the primary source of *mcr-1* gene isolates [1]. Among recipient cells, the frequency of *mcr-1* gene mobility ranges from 10^−1^ to 10^−3^ [65]. *Vibrio parahaemolyticus*, a non‐enterobacteriaceae, has also been shown to carry the *mcr-1* gene [127].
ii. *mcr-2* gene: The *mcr-2* gene is prevalent in *E. coli* from pigs and cows [1]. Thirty‐three variants have been discovered, and the size of the *mcr-2* gene is 1617 bp, located on the IncX4 plasmid [128]. The *mcr-2* gene co‐harbours with the lipid phosphatase gene and shares 76.74% of its similarities with the *mcr-1* gene [129].
iii. *mcr-3* gene: *Escherichia coli* isolates from pigs were the first to be identified with the *mcr-3* gene [66]. Approximately 1626 bp make up the *mcr-3* gene, and 30 variants have been found [130, 131]. The *mcr-3* gene exhibits 47% and 45% nucleotide similarity with the *mcr-2* and *mcr-1* genes, respectively [66]. The *mcr-3* gene is often spread by transposons rather than plasmids and co‐located with *mcr-1* and *mcr-3.6.* Additionally, it co‐harbours with ARGs (*qnrS1* and *blaCTX-M-55*) [132].
iv. *mcr-4* gene: *Salmonella enterica* from Italian pigs was initially found to contain the *mcr-4* gene [67]. Six variants of the *mcr-4* gene have been identified, and the size of the *mcr-4* gene is 1626 bp [133]. The *mcr-4* gene exhibits 34%, 35% and 49% amino acid sequence similarity with MCR‐1, MCR‐2 and MCR‐3, respectively. In Spain, the *mcr-4* gene is quite prevalent [1].
v. *mcr-5* gene: German researchers first identified the *mcr-5* gene in *Salmonella paratyphi* in poultry [128]. The *mcr-4* gene, which belongs to the Tn3 family, has a size of 1644 bp, and four variants have been identified [1]. Protein of the *mcr-5* gene exhibits 36.11%, 35.29%, 34.72% and 33.71% amino acid sequence similarity with MCR‐1, MCR‐2, MCR‐3 and MCR‐4, respectively [134]. Widespread dispersion is indicated by the fact that 82% of plasmids containing the *mcr-5* gene can undergo conjugation [118].
vi. *mcr-6* gene: The host in which the *mcr-6* gene was first found, *Moraxella pluranimalium* [135]. Only one variant of the *mcr-6* gene has been detected in clinical and environmental isolates [136]. The size of the *mcr-6* gene is 1617 bp and contains phosphoethanol‐lipid‐A transferase activity [1].
vii. *mcr-7* gene: China was the first country to identify the *mcr-7* gene in *K. pneumoniae* from chicken [115]. It exhibits 70% amino acid sequence similarity with MCR‐3 and is found to co‐harbour with other ARGs [1]. Only one variant of the *mcr-7* gene has been identified in clinical and environmental isolates [115].
viii. *mcr-8* gene: It was first identified in China from pig faeces and human sputum from *K. pneumoniae* [113]. Five variants have been identified, and the size of the *mcr-8* gene is 1698 bp [1]. It exhibits 31.08%, 30.26%, 39.96%, 37.85%, 33.51%, 30.43% and 37.46% amino acid sequence similarity to MCR‐1, MCR‐2, MCR‐3, MCR‐4, MCR‐5, MCR‐6 and MCR‐7, respectively [137]. The *mcr-8* gene is situated on a stable plasmid and is found to co‐harbour with the *mcr-3 (mcr-3.21, mcr-3.26* and *mcr-3.28*) and *mcr-4 (mcr-8.4*) genes [1, 138]. The *mcr-8* gene and ARGs are co‐located, and the plasmid containing both genes spreads quickly throughout *Enterobacteriaceae* [1].
ix. *mcr-9* gene: American patients were the first to isolate the *mcr*‐9 gene from *Salmonella enterica* [1]. The *mcr-9* gene has a high amino acid sequence similarity with MCR‐3, MCR‐7 and MCR‐4, and 64.5% with the *mcr-3.17* allele [139]. It coexists with MDR *Enterobacteriaceae* in human blood and sputum with *blaNDM-1* and *blaVIM-4*, respectively [140]. Only three variants of the *mcr-9* gene have been identified [1].
x. *mcr-10* gene: It was identified from *Klebsiella pneumonia* ST11 strain isolates in clinical settings [1]. Screening of the bacterial GeneBank library of bacterial genomes proved that some bacteria harbour the *mcr-10* gene. The *mcr-10* gene is co‐located with the *mcr-8* gene [141]. Reduced susceptibility was shown by the *mcr-10* and *mcr-8* genes to colistin and tigecycline, indicating sporadic dissemination across the globe [142]. One investigation revealed that the *mcr-10* gene is situated on a novel plasmid termed IncFIB that is carried by *E. coli* [143].

## 5. Strategies to Combat Colistin Resistance

**FIGURE 4:**
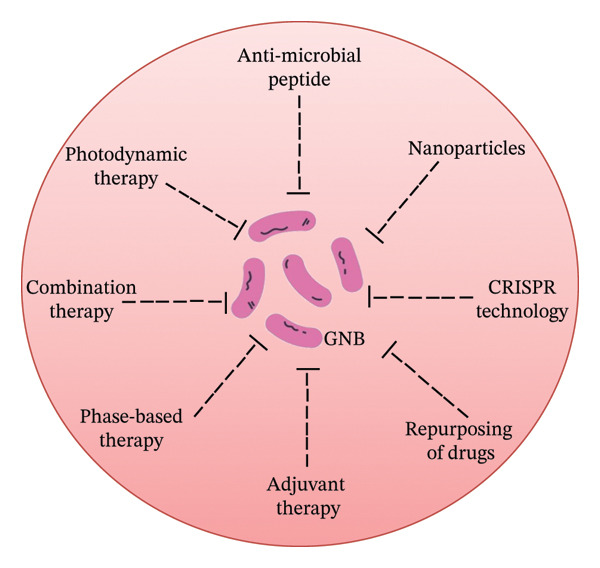
Strategies to combat colistin‐resistant strains.

The rapid spread of colistin‐resistant bacteria and the limitation of available treatments raise concerns about the threat to global health. Combination therapy, drug repurposing, adjuvant therapy, antimicrobial peptides, photodynamic treatment, nano‐therapeutics, bacteriophage‐based therapy and CRISPR technology are potential treatments available so far (Figure 4).

i. Combination therapy: Combination therapy utilizes a combination of colistin with other antibiotics to manage GNB‐threatened infections. Colistin with either tobramycin or ciprofloxacin works well together to combat *A. baumannii* [144]. Among various combinations, colistin with imipenem, aztreonam, vancomycin and ceftazidime are the best combinations that exhibit higher potency against GNB bacteria that are resistant to colistin [145]. Studies also showed that the combination of fusidic acid with colistin exhibits promising therapeutic efficacy against colistin‐resistant strains [146, 147]. PYED (pregnadiene‐11‐hydroxy‐16,17‐epoxy‐3,20‐dione)‐1 exhibits anti‐microbial and anti‐biofilm efficacy and shows promising anti‐microbial effects against MDR‐*A. baumannii* with colistin [148]. A clinical study observed promising treatment efficacy of the combination of colistin with rifampicin and vancomycin against ventilator‐associated pneumonia (VAP) patients [149]. In clinical settings, colistin is frequently employed in conjunction with gentamicin, fosfomycin, meropenem or tigecycline [150].
ii. Drug repurposing: Drug repurposing refers to the utilization of existing drugs to treat colistin‐resistant strains in clinical settings. Several clinical investigations demonstrated that oxyclozanide, closantel and niclosamide enhanced the effectiveness of colistin against GNB strains [151–154]. Tavaborole, an antifungal agent, exhibited promising therapeutic efficacy against MDR strains [155]. Antipsychotic (fluspirilene), anticarcinogenic (5‐fluorouracil and mitomycin‐C) and anti‐κB kinase drugs exhibit promising antibacterial effects against MDR‐*A. baumannii* [156, 157]. A metabolite of the anti‐carcinogen N‐desmethyltamoxifene significantly improves the condition of patients infected with *Escherichia coli* and *A. baumannii* when combined with colistin [158]. Patients infected with colistin‐resistant *Enterobacteriaceae* strains significantly improved with PFK‐158 (an anti‐carcinogenic drug) [159]. A recent study showed that shikonin, a natural remedy, enhanced the effectiveness of colistin [160]. One clinical study demonstrated that valnemulin, an FDA‐approved medication, improved colistin’s therapeutic efficacy against MDR‐GNB strains in a synergistic manner [161].
iii. Adjuvants: Adjuvants of non‐antibiotic molecules refer to adjuvant therapy that improves the therapeutic efficacy of antibiotics by reducing drug toxicity and drug expulsion, enhancing membrane permeability, altering microbial metabolism and iron homeostasis, membrane disruption and inhibition of biofilm formation and lipid‐A modification [162]. Several studies reported various adjuvant compounds (kaempferol, nitazoxanide, 2‐aminoimidazole, chrysin, curcumin, panduratin‐A, IMD‐0354, auranofin, disulfiram and capsaicin) that utilized one of these strategies to enhance the therapeutic efficacy of colistin [163–172].
iv. Peptides: Peptides with wide‐spectrum efficacy are called anti‐microbial peptides that open the window for effective treatment of MDR, XDR and colistin‐resistant strains [173, 174]. Several investigations reported that various anti‐microbial peptides (NuriPep 1653, mastoparan, Esc (1‐21), Ω76, melittin, LS‐stomoxyn, 2 K4L, LS‐sarcotoxin, indolicidin and Cec4), either alone or in combination with colistin, exhibit therapeutic efficacy against colistin‐resistant strains through variable mechanisms [175–181].
v. Photodynamic therapy: Photodynamic therapy is an emerging novel technology that inactivates a wide range of pathogens [182]. One investigation experimented on photodynamic therapy and reported that toluidine blue‐O (TBO) and light‐emitting diode (LED) inactivated the PmrA/pmrB systems in *A. baumannii* [183]. Another study observed that TBO and LED permit antibiotic penetration inside colistin‐resistant strains through up‐regulation of the *ompA* gene [184]. Synergistically, photodynamic therapy reduces MIC by up to 11 times, increasing the therapeutic efficacy of colistin [185].
vi. Nanoparticles (NPs): NPs are a novel strategy to combat colistin‐resistant strains that induce mutation via various mechanisms that may or may not operate simultaneously. However, the exact mechanisms behind NP‐mediated bacterial cell death are still unexplored. Research reported that NPs disrupt the membrane potential on binding to the outer bacterial surface and interact with various bacterial cell components, leading to oxidative stress, enzyme inhibition, perturbation of electrolyte homeostasis and gene expression, which eventually results in cell lysis [186]. Investigation on AgNPs found that NPs prevent biofilm formation and mitigate gene expression (*csuA/B*, *bap* and *OmpA*) linked to biofilm formation in *A. baumannii* [187]. ZnO NPs also mitigate biofilm formation by reducing genes (*abeM*, *adeC* and *adeA*) linked to efflux pumps [188]. Multiple investigations have found that AgNPs enhance the therapeutic potency of colistin, as well as decrease the MIC in a synergistic manner [189]. Within 4 h of using Se NPs with colistin, notable decreases in bacterial load were noted [190].
vii. Bacteriophage‐based therapy: The scientific community is increasingly using bacteriophage‐based therapy to treat drug‐resistant strains [191]. Colistin and PMK34 bacteriophage lysine lower the MIC by 32 times and enhance the susceptibility of colistin‐resistant GNB strains in 50% human serum, as well as in Mueller–Hinton broth [192]. Colistin and the phage vWU2001 combination showed a reduction in bacterial growth and increased bacterial clearance as compared to single therapy, which indicates a synergistic effect against colistin‐resistant strains [193]. One investigation on *A. baumannii* demonstrated that a lytic phage named *IsfAB78* mitigates biofilm formation by 87% [194]. In a clinical investigation, the condition of elderly diabetic patients with necrotic pancreatitis who did not respond to the final line of antibiotics (tigecycline and colistin) improved after receiving treatment with nine bacteriophages [194]. Additional improvement has been observed in patients with the addition of minocycline for 5 days with bacteriophage therapy against phage‐resistant *A. baumannii* [195].
viii. CRISPR‐based therapy: A molecular gene‐editing method called CRISPR technology is the potential hope for the treatment of colistin‐resistant strains. CRISPR–Cas systems mitigate colistin resistance by ROS production, inhibition of biofilm formation and reduction of efflux pump activity [196]. Removal of plasmid‐containing *mcr* genes from *E. coli* isolates results in enhanced susceptibility to colistin. Successful eradication of plasmids has been achieved from 14EC033a and 14EC007 through CRISPR‐Cas9 [197]. Deletion of multiple gene copies can be easily achieved using a single sgRNA with CRISPR technology [197]; however, the procedure must be carefully managed to avoid undesired genetic changes.
ix. Immunotherapeutic approaches: Monoclonal, bispecific antibodies and vaccines are potential alternatives employed to combat drug‐resistant infections [198]. A focused review emphasized the role of LPS as a potential target antigen for the development of vaccines against GNB [199]. DNA‐delivered monoclonal antibodies (DMAbs) have demonstrated protection against a *P. aeruginosa* challenge in a mouse model [200]. A bispecific antibody (MEDI3902) that targets the polysaccharide of *P. aeruginosa* has been shown to inhibit biofilm formation [201].

## 6. Conclusion and Future Direction

The rapid emergence of colistin‐resistant strains occurs due to excessive antibiotic use, alternative drug scarcity, horizontal gene transfer, tourism and other potential mechanisms that induce high mortality as well as morbidity, and become a global health concern. The mechanisms contributing to colistin resistance include plasmid‐harbouring mcr genes, chromosomal genes, biofilm formation and hetero‐resistance. Various treatment strategies have been developed to treat colistin‐resistant strains. A combination of different treatment strategies must be exploited to prevent the rapid emergence of colistin‐resistant strains. Researchers should concentrate on developing bio‐hybrid magnetic micro‐robots that are resistant to colistin by utilizing non‐antibiotic molecules. Additionally, researchers must also focus on clinical toxicity and the issue of restricted drug penetration into different organs or tissues regarding various treatment strategies. Antibiotic policies in clinical, agricultural and veterinary settings need to be revisited for any amendments. Collaborative research efforts must be carried out to secure the future of modern medicine and to reduce AMR‐driven morbidity and mortality in healthcare sectors.

## Author Contributions

D.S. conceived the idea. I.S., A.K., I.K.V., D.S. and A.K.S. wrote the initial draft and prepared the figures. All authors reviewed the final manuscript.

## Funding

The authors have nothing to report.

## Ethics Statement

The authors have nothing to report.

## Consent

The authors have nothing to report.

## Conflicts of Interest

The authors declare no conflicts of interest.

## Data Availability

Data sharing is not applicable to this article as no new data were created or analysed in this study.
